# Trophic transfer and individual impact of nano-sized polystyrene in a four-species freshwater food chain

**DOI:** 10.1038/s41598-017-18849-y

**Published:** 2018-01-10

**Authors:** Yooeun Chae, Dokyung Kim, Shin Woong Kim, Youn-Joo An

**Affiliations:** Department of Environmental Health Science, Konkuk University, 120 Neungdong-ro, Gwangjin-gu, Seoul, 05029 Korea

## Abstract

This study investigated the trophic transfer, individual impact, and embryonic uptake of fluorescent nano-sized polystyrene plastics (nanoplastics) through direct exposure in a freshwater ecosystem, with a food chain containing four species. The alga *Chlamydomonas reinhardtii*, water flea *Daphnia magna*, secondary-consumer fish *Oryzias sinensis*, and end-consumer fish *Zacco temminckii* were used as test species. In the trophic transfer test, algae were exposed to 50 mg/L nanoplastics, defined as plastic particles <100 nm in diameter; higher trophic level organisms were exposed through their diet. In the direct exposure test, each species was directly exposed to nanoplastics. Microscopic analysis confirmed that the nanoplastics adhered to the surface of the primary producer and were present in the digestive organs of the higher trophic level species. Nanoplastics also negatively affected fish activity, as measured by distance traveled and area covered, and induced histopathological changes in the livers of fish that were directly exposed. Additionally, nanoplastics penetrated the embryo walls and were present in the yolk sac of hatched juveniles. These observations clearly show that nanoplastics are easily transferred through food chain, albeit because of high experimental dosages. Nevertheless, the results strongly point to the potential health risks of nanoplastic exposure.

## Introduction

Every year, enormous amounts of plastics are produced and discarded into the aquatic environment^1–5^; these are slowly eroded and weathered into small particles by physical, chemical, and biological processes^6,7^ and can accumulate in aquatic environments, including the sea^1^, shorelines^3^, estuaries^8^, beach sediments^9^, lakes^10,11^, and freshwater ecosystems^12^. Plastic particles <5 mm in diameter are called microplastics and those <100 nm in diameter are called nanoplastics^1,13,14^. Studies on plastic litter in the oceans date to the 1970s^15–19^. Serious concerns about micro- and nanoplastics in aquatic ecosystems arose in the 2000s, because of their abundance in marine ecosystems^1,19–24^ and discovery in the bodies of marine organisms^25–30^.

Micro- and nanoplastics may induce various toxic and adverse effects in aquatic organisms^31–39^. Several studies have reported that microplastics can carry contaminants, such as polycyclic aromatic hydrocarbons (PAHs), polychlorinated biphenyls (PCBs), perfluoroalkyl acids (PFAA), persistent organic pollutants (POPs), pharmaceuticals, personal care products (PPCPs), and metals, into aquatic media because of their physicochemical properties^5,40–43^. Plastics contain not only polymers but also additive chemicals such as plasticizers, antioxidants, UV stabilizers, and flame retardants, which could be released into aquatic environments^44–46^, causing harm to aquatic organisms^47–49^. These contaminants may be transferred through the food chain to predators at upper trophic levels^46,50–52^. Their presence poses a considerable threat to aquatic ecosystems, the health of aquatic organisms, and human health. Therefore, the fate and behavior of micro- and nanoplastics in ecosystems, and their transfer between organisms and from organisms to the environment, must be examined.

In the present study, we chose fluorescent nano-sized polystyrene (nPS) as the model nanoplastic material whose transfer along the food chain is readily observable in laboratory tests. Using this material, we investigated the trophic transfer of this fluorescent nPS in a freshwater ecosystem, through a food chain consisting of four species, and recorded the effects of this material on aquatic organisms. We assumed that nanoplastics can be transferred to higher trophic level aquatic organisms and may negatively affect their health via dietary exposure (through food ingestion) and direct exposure (every form of contact, including food ingestion). The main goal of this study was to assess the transfer of these particles from freshwater algae, the primary producer, to carnivorous fish, the end consumer. In addition, we assessed the effects of nanoplastics on each aquatic organism.

## Results

### Characteristics of nPS

The size distributions, zeta (ζ)-potentials, and electron microscope images of nPS particles are shown in Fig. S1. Average diameters of nPS particles were 60.39, 57.45, and 57.29 nm in distilled water (DW), moderately hard water (MHW), and tris-acetate-phosphate medium (TAP), respectively. ζ-Potentials of nPS in each medium were −42.1, −17.4, and −14.1 mV in DW, MHW, and TAP, respectively. Mean diameters of nPS in DW and each test medium varied slightly, but the particles were rarely aggregated. The absolute values of ζ-potentials declined in test media, indicating that nPS particles are less stable in MHW and TAP.

### Visual evidence of nPS trophic transfer

**Figure 1:**
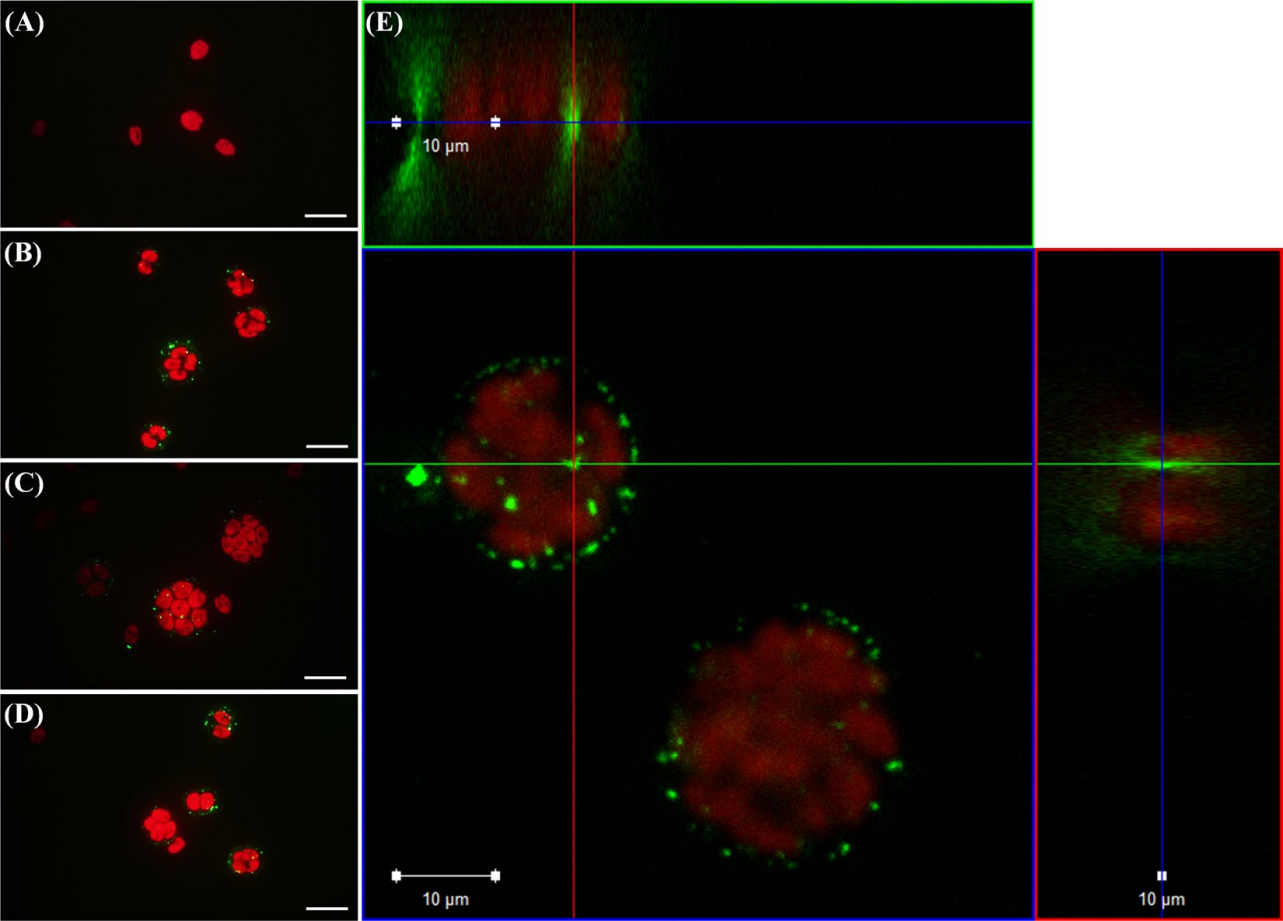
Observation via optical microscopy (**a**–**d**) and confocal laser scanning microscopy (**e**) of the alga *Chlamydomonas reinhardtii* (red emissions) directly exposed to nano-sized polystyrene (nPS; green emissions) for 72 h. Scale bar = 20 (**a**–**d**) and 10 μm (**e**).

Due to direct exposure, nPS attached to the surface of *C*. *reinhardtii* (Fig. 1). Unlike the control group (Fig. 1a), the exposed groups (Fig. 1b–d) showed clearly separated red (auto-fluorescence of algae) and green (nPS fluorescence) emissions. Via confocal laser scanning microscopy (CLSM), we confirmed that nPS penetrated the outer layer of *C*. *reinhardtii* during cell division and attached to the surface of zoospores (Fig. 1e).

**Figure 2:**
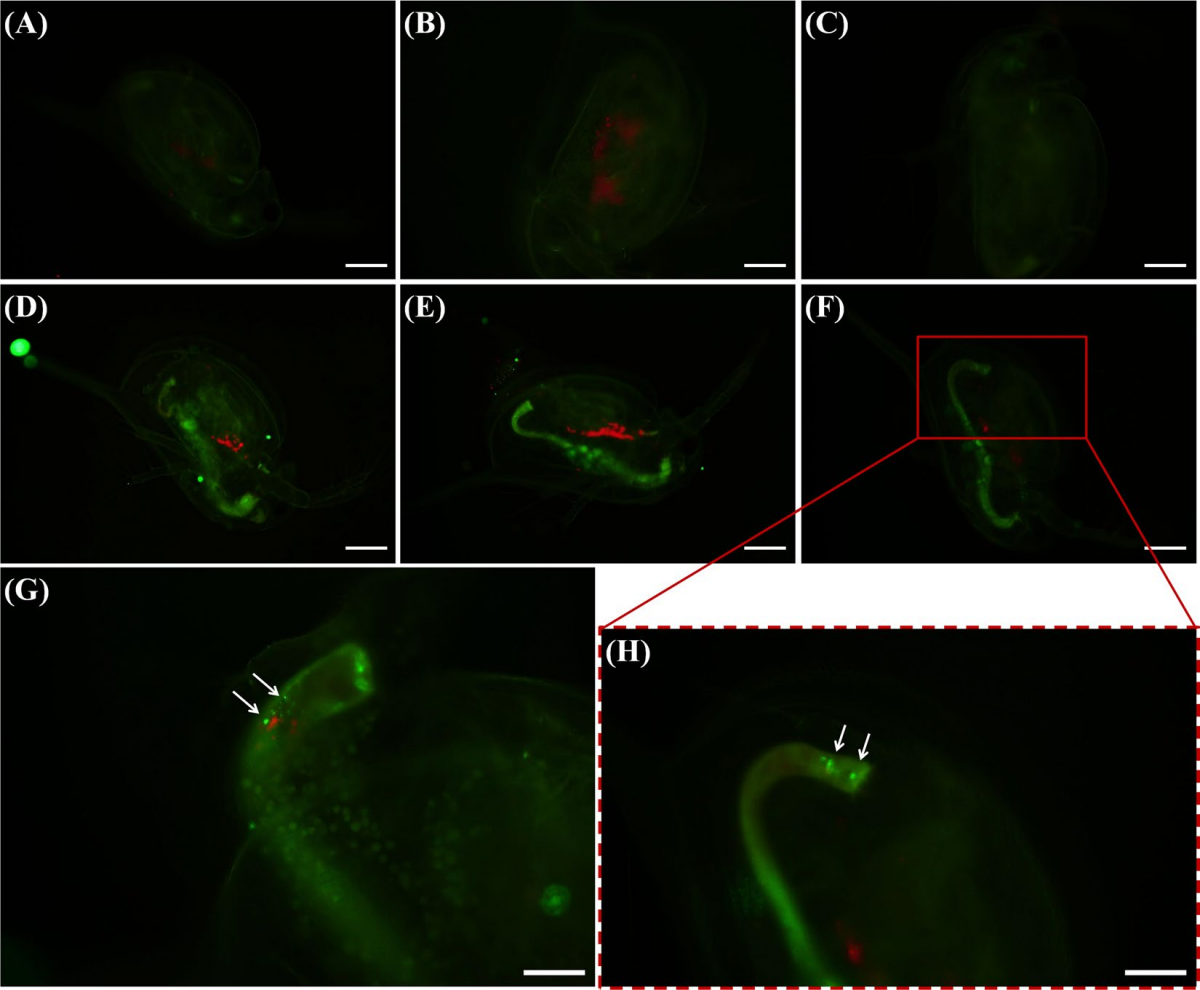
Green fluorescence of nano-sized polystyrene (nPS) in *Daphnia magna* that had consumed *Chlamydomonas reinhardtii* (red fluorescence). Control groups (**a**–**c**), exposed groups (**d**–**h**), and expanded pictures of exposed individuals (**g**,**h**). Scale bar = 200 (**a**–**f**) and 100 μm (**g**,**h**).

**Figure 3:**
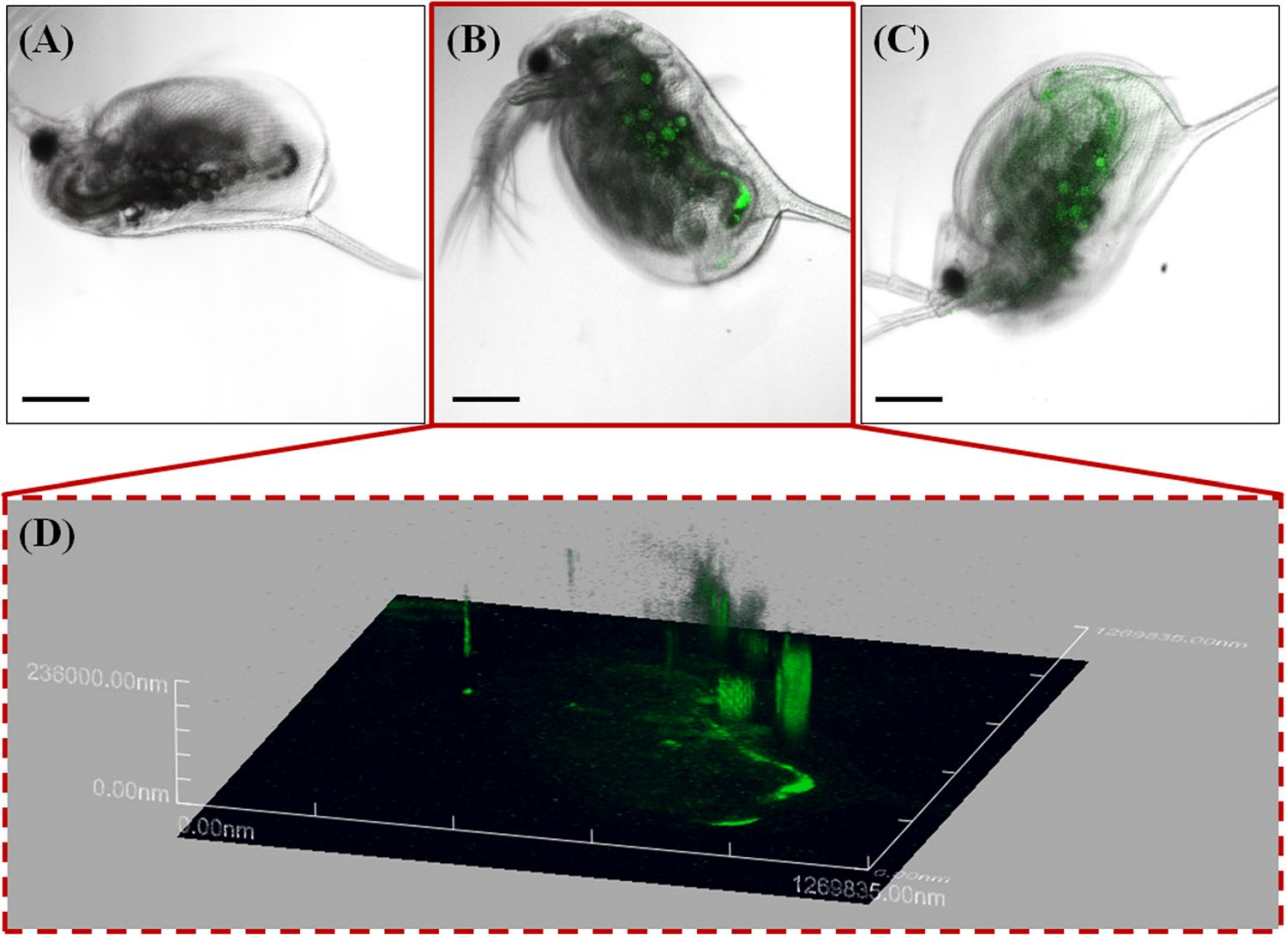
Confocal laser scanning microscope (CLSM) images of *Daphnia magna* that were not exposed to nano-sized polystyrene (nPS; green emissions) (**a**) and that were exposed through the diet (**b**,**c**). Z-stack image of individual exposed through the diet (**b**), observed using CLSM (**d**). Z-stack image (**d**) provides evidence of nPS uptake through dietary exposure of *D*. *magna*. Green fluorescent nPS was observed in the gut of exposed *D*. *magna*. Scale bar = 200 μm.

**Figure 4:**
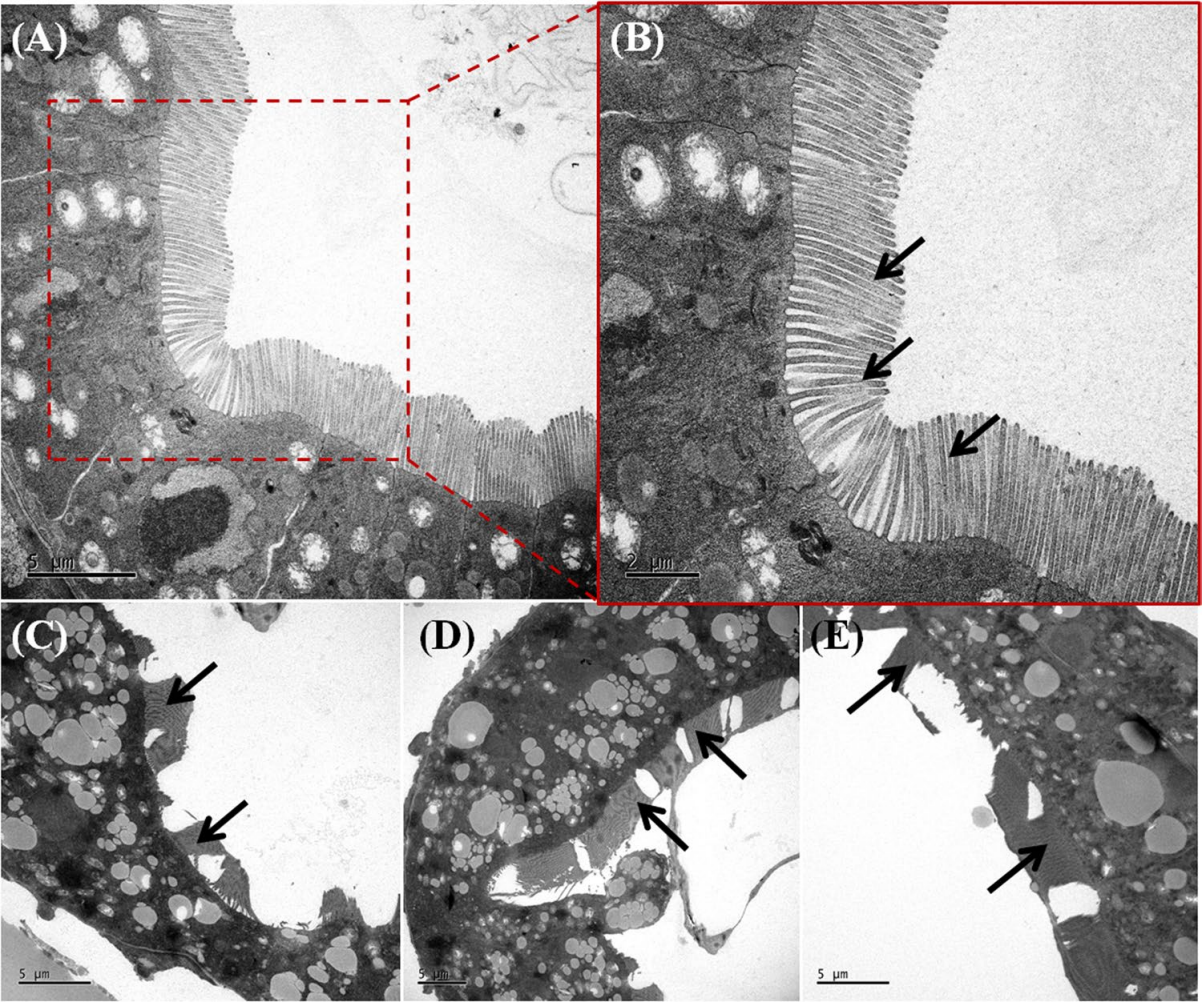
Transmission electron microscope (TEM) images of the transverse sectioned gut wall and microvilli (black arrows) in *Daphnia magna*. The guts and microvilli of *D*. *magna* that consumed non-exposed algae (**a**,**b**) and algae that were exposed to nano-sized polystyrene (nPS) (**c**–**e**) were observed. (**a**) Scale bar = 5 (**a**,**c**–**e**) and 2 μm (**b**).

The attached nPS evidently transferred to *D*. *magna* through filter feeding and was observed in the gut of *D*. *magna* under the microscope (Fig. 2). Compared with control groups (Fig. 2a–c), exposed groups (Fig. 2d–h) showed distinct green fluorescence in their guts. Aggregated nPS was found at the terminal end of the guts of *D*. *magna* (Fig. 3g,h). We analyzed *D*. *magna* via CLSM and used Z-stack imaging to confirm the presence of nPS in the inner guts. Figure 3 compares the CLSM analysis of control individuals (Fig. 3a) and exposed individuals (Fig. 3b,c) and shows the Z-stack image of the exposed individual from Fig. 3b (Fig. 3d). We also used bio-transmission electron microscopy (Bio-TEM) to analyze the gut and microvilli of *D*. *magna* that were exposed through the diet to *C*. *reinhardtii* contaminated with nPS (Fig. 4). Control group *D*. *magna* were undamaged (Fig. 4a,b), whereas exposed *D*. *magna* had squashed and torn-out microvilli (Fig. 4c–e). This visual evidence confirms that nPS enters the guts and causes histological damage to the intestinal walls of *D*. *magna* through trophic transfer.

**Figure 5:**
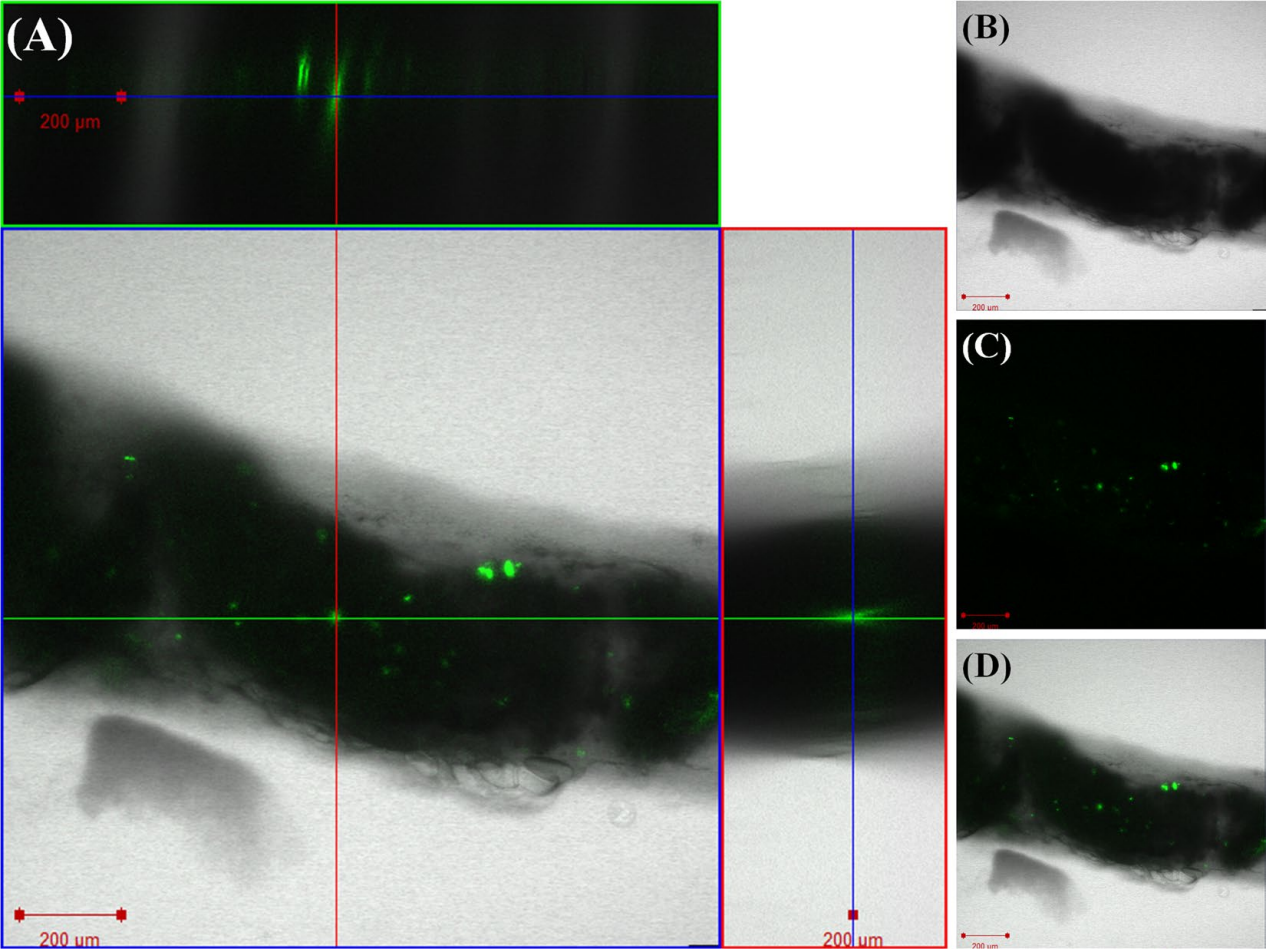
Confocal laser scanning microscope (CLSM) images of *Oryzias sinensis* intestines after the fish consumed *D*. *magna* that were exposed to nano-sized polystyrene (nPS; green emissions). Z-stack images (**a**) confirmed the presence of nPS in the intestines of *O*. *sinensis*, as did merged CLSM images of optical image (**b**), fluorescent-only image (**c**), and merged image (**d**). Scale bar = 200 μm.

At the next level of dietary exposure, there was no fluorescence in the gut of control fish (Fig. S2a,b) but the green fluorescence of nPS was observed in the guts of exposed *O*. *sinensis* (Fig. S2c,d). To confirm that nPS entered the gut of *O*. *sinensis*, the extracted intestines of *O*. *sinensis* were also observed via CLSM (Fig. 5). The green fluorescence of nPS was clearly observed in the intestines, confirming the entry of nPS into the intestine via the food chain (Fig. 5a). In addition, the fluorescence of nPS in the feces of *O*. *sinensis* was observed under optical microscope with a fluorescent filter (Fig. S3).

**Figure 6:**
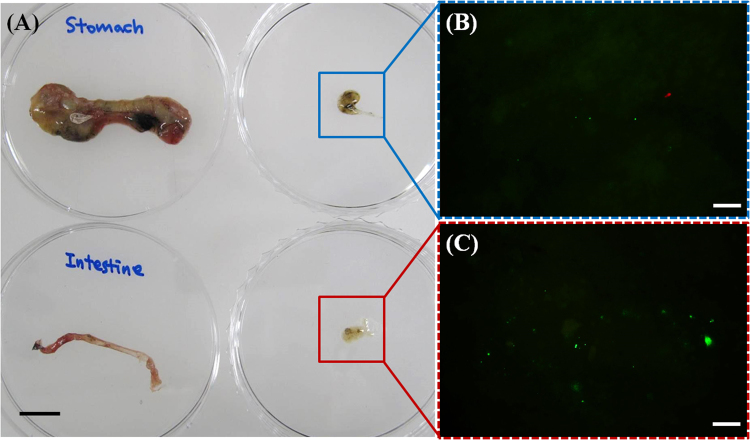
The organs (stomach and intestines) and their contents (**a**) of *Zacco temminckii* exposed through the diet to nano-sized polystyrene (nPS; green emissions) and the fluorescence of nPS in the organ contents (**b**,**c**). Red fluorescence is auto-fluorescence of *Chlamydomonas reinhardtii* and green fluorescence is that of nPS. Scale bar = 1 cm (**a**) and 20 μm (**b**,**c**).

The final trophic transfer of nPS from *O*. *sinensis* to *Z*. *temminckii* was observed through an analysis of the extracted stomach and intestines of *Z*. *temminckii*. As shown in Fig. 6, the stomach and intestines from exposed *Z*. *temminckii* contained nPS from the consumed *O*. *sinensis*. More nPS was observed in the intestines (Fig. 6c) than in the stomach (Fig. 6b). There were also differences in fluorescence in the feces of control and exposed fish (Fig. S4). Under an optical microscope with a fluorescent filter, (Fig. S4a), the feces of exposed fish had brighter green fluorescence than the feces of control fish (Fig. S4a), indicating an aggregation of nPS (Fig. S4b).

### Toxic effects of nPS on test species

The results of individual toxicity tests on *C*. *reinhardtii* and *D*. *magna* are shown in Fig. S2. There was little or no mortality or effective toxicity on *C*. *reinhardtii* (Fig. S5a) and *D*. *magna* (Fig. S5b) in the range of tested concentrations for 72 h and 48 h, respectively (*p* > 0.05).

**Figure 7:**
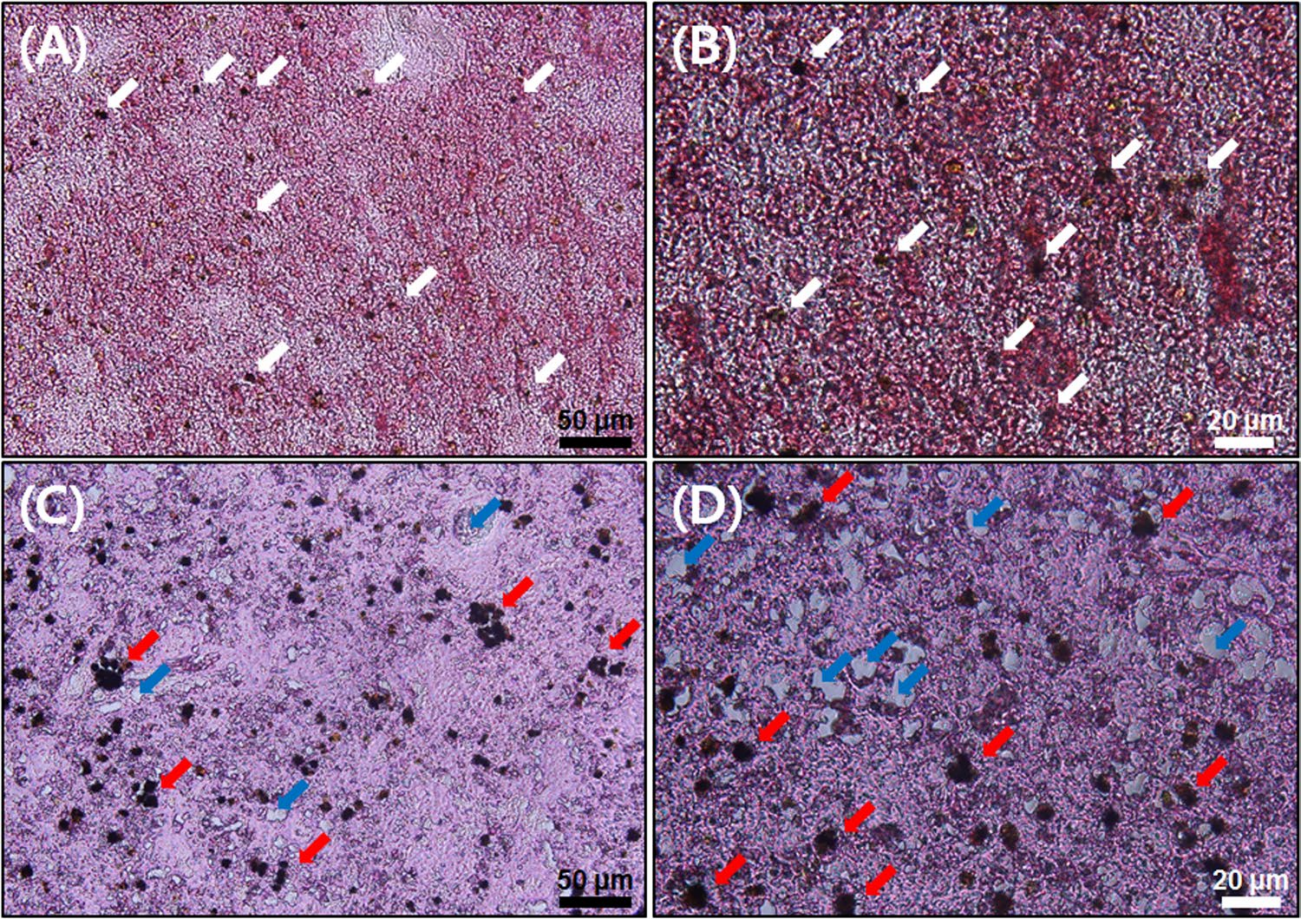
H&E (hematoxylin and eosin) stained liver tissues from *Zacco temminckii* not exposed (**a**,**b**) and exposed (**c**,**d**) to nano-sized polystyrene (nPS). Normal nuclei (white arrow), damaged and aggregated nuclei (red arrow), and vacuole (blue arrows) in liver tissue. Scale bar = 50 (black) and 20 (white) μm.

However, nPS caused several abnormalities in both *O*. *sinensis* and *Z*. *temminckii* after direct exposure for 7 d. First, livers from non-exposed and exposed *Z*. *temminckii* showed different histological patterns (Fig. 7). The livers from the control group had regularly distributed stained dark nuclei (Fig. 7a,b), but the livers of the exposed group had aggregated and agglomerated nuclei (Fig. 7c,d). This finding indicates that cells in liver tissues from exposed *Z*. *temminckii* were broken and destroyed. There were also several vacuoles in the liver tissues of exposed *Z*. *temminckii*. Synthetically, direct exposure of *Z*. *temminckii* to nPS for 7 d caused structural and histopathological problems. There was a slight increase in the total amount of cholesterol in the blood serum of exposed *Z*. *temminckii* compared with non-exposed fish (Fig. S6). There were slight increases of total cholesterol in nPS-exposed fish, but because there were insufficient replicates, these data could not be statistically analyzed.

**Figure 8:**
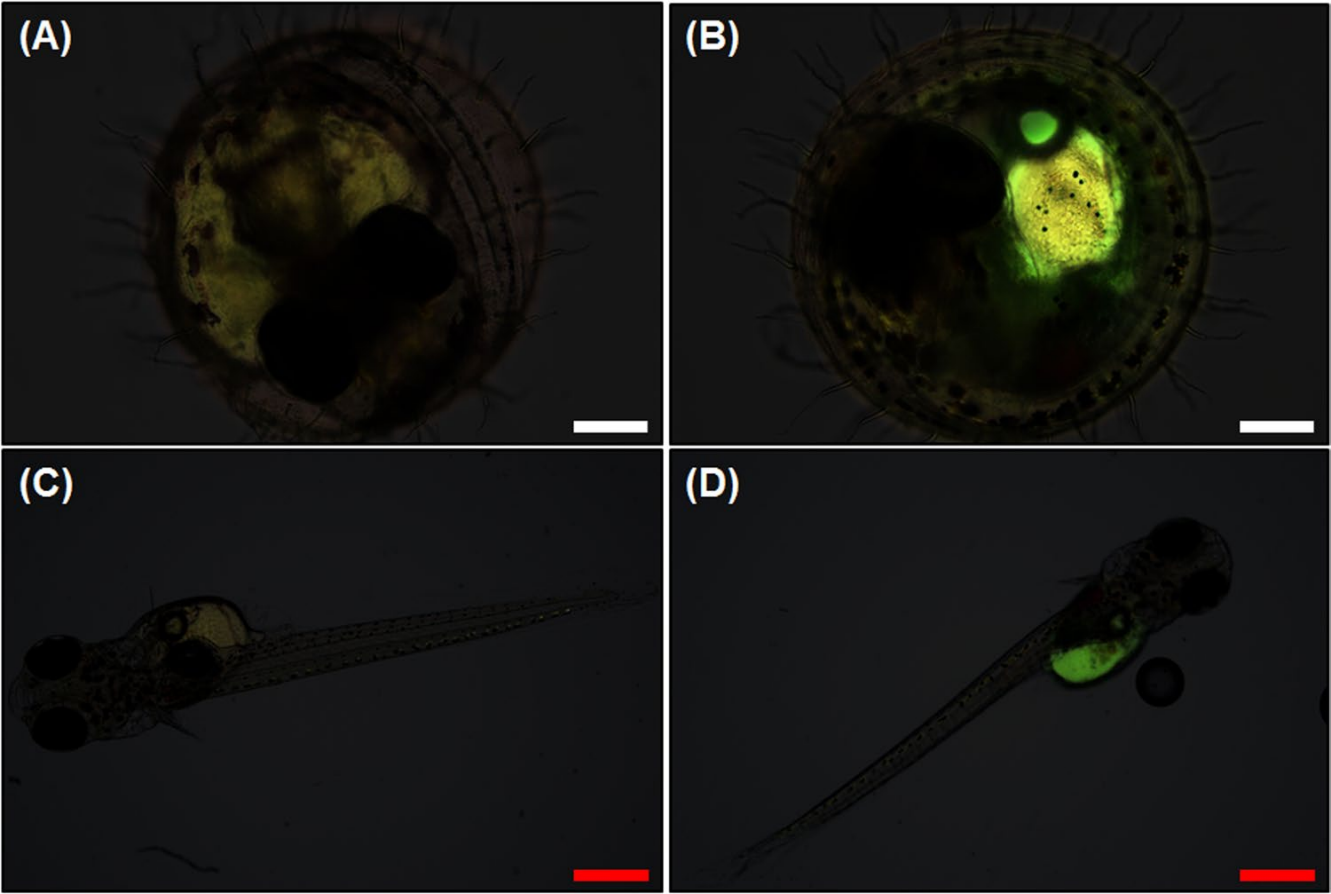
Merged (bright field and fluorescence) images of embryos and juveniles of *Oryzias sinensis* that were not exposed (**a**,**c**) and that were exposed (**b**,**d**) to nano-sized polystyrene (nPS). The embryos are 144 hours post fertilization (hpf) and juveniles are observed <24 h after hatching. The bright green fluorescence (**b**,**d**) indicates the uptake of nPS in organisms. Scale bar = 200 (**a**,**b**, white) and 500 (**c**,**d**, red) μm.

When embryos were directly exposed to 5 mg/L nPS, the same concentration to which adult *O*. *sinensis* were exposed, they showed bright green fluorescence, as did the yolk sacs of exposed embryos (Fig. 8). Compared with the embryos (Fig. 8a) and hatched juveniles (0 d) (Fig. 8c) from the control group, nPS-exposed embryos (Fig. 8b) and juveniles (0 d) (Fig. 8d) showed clear nPS fluorescence in the yolk sac.

**Figure 9:**
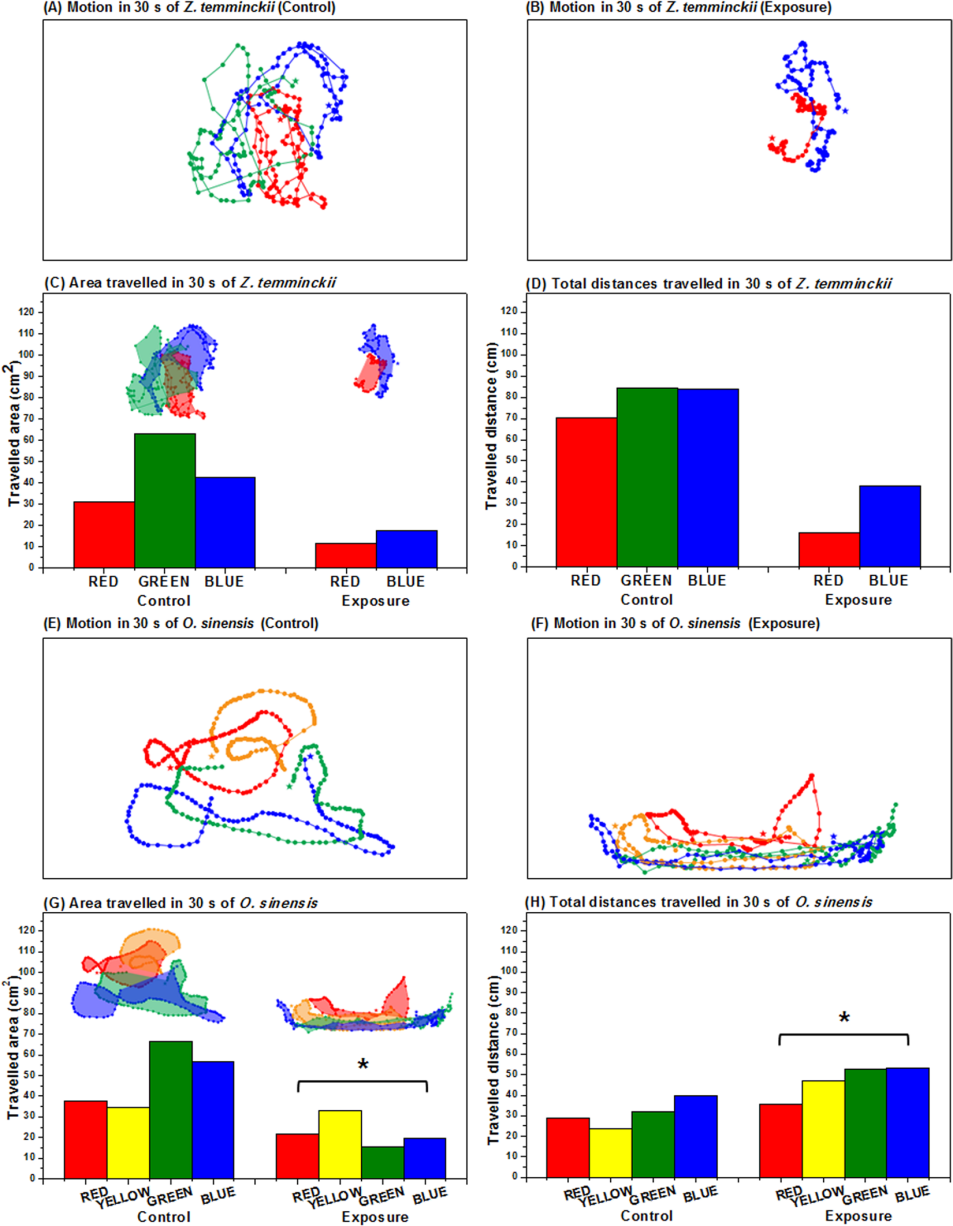
The locomotive activities of *Zacco temminckii* (**a**–**d**) and *Oryzias sinensis* (**e**–**h**). Each bar in graphs shows the activity of one individual. Motion was recorded for 30 s of *Z*. *temminckii* not exposed to nPS (**a**) and exposed to 5 mg/L nano-sized polystyrene (nPS) (**b**). Quantitative data of the area traveled (**c**) and the total distances covered (**d**) for 30 s of nPS non-exposed and exposed *Z*. *temminckii*. Motion of *O*. *sinensis* not exposed (**e**) and exposed to nPS (**f**) was recorded for 30 s. Quantitative data of the area traveled (**g**) and the total distances covered (**h**) for 30 s of *O*. *sinensis* not exposed and exposed to nPS (*p* < 0.05). Different colors in each box designate different individuals, and the dots and the lines represent the locations for every 0.3 s and the movements of each individual, respectively.

In addition, locomotive activities of *O*. *sinensis* and *Z*. *temminckii* were affected by nPS exposure (Fig. 9). The area through which fish traveled was significantly smaller in the nPS-exposed groups of both fish species (Fig. 9c,g). Total distances traveled in 30 s were significantly shorter in nPS-exposed *Z*. *temminckii* (Fig. 9d), whereas in nPS-exposed *O*. *sinensis*, they were slightly longer (*p* < 0.05) (Fig. 9h). These differences may be due to different swimming patterns between the two test species. Comparisons of free-swimming patterns and distances between individuals in the control group (Fig. 9a,e) showed that nPS-exposed fish maintained shorter distances between each individual and swam in shoals (Fig. 9b,f).

## Discussion

We investigated various negative effects of nPS on freshwater species and confirmed that nPS can induce disturbances in the morphology of liver tissue, lipid metabolism, embryos, and locomotive activities of fish. These results lead us to conclude that nanoplastics have adverse effects on aquatic organisms and induce biochemical, behavioral, and histological changes. Several previous studies have assessed the histopathological changes in the livers of fish fed micro- or nanoplastics. Rochman *et al*.^53^ and Lu *et al*.^54^ also confirmed that plastic particles can change and disturb liver morphology and induce histopathological changes in the liver tissue. These results indicate that nanoplastics might act as contaminants or pollutants in the bodies of fish and accumulate in the liver, the organ that detoxifies the body. Von Moos *et al*.^31^ confirmed disturbances of lipid metabolism in mussels exposed to microplastics, and Cedervall *et al*.^46^ investigated metabolic (cholesterol) changes in *Carassius carassius* exposed to nPS through their diet. Similarly, in the present study, we analyzed the differences in the total amount of cholesterol between control fish and nPS-exposed fish but could not carry out the statistical analysis because there were too few replicates. However, we confirmed that total cholesterol in fish slightly increased after nPS exposure. These results suggest that long-term exposure to nanoplastics may cause nutritional and health problems in fish and other organisms. Regarding exposure to embryos, Manabe *et al*.^55^ also observed bright green fluorescence in embryos exposed to fluorescent latex particles with a diameter of 50 nm. In another study, fluorescent nPS (diameters of 40 and 50 nm) entered the embryos of sea urchins^14^. Lee *et al*.^56^ confirmed that the pore size of embryo chorions of zebrafish was 0.5–0.7 μm, which is much larger than the diameter of nPS used in the present study. Although the test species that we used was *O*. *sinensis*, previous research can still be applicable. The results of previous studies, combined with our results, indicate that small plastics with nano-sized diameters can penetrate the walls of embryos and accumulate in the lipid of embryos due to the hydrophobic property of nanoplastics, the porous membranes of embryos, and material exchanges between organisms and environments. Mattsson *et al*.^51^ reported that nPS-exposed fish occupied less space in aquaria than non-exposed fish. Cedervall *et al*.^46^ observed that nPS-exposed fish moved more slowly than control fish and attributed it to nPS transfer from food to several organs, including the brain, via the bloodstream. These transferred nano-sized particles can cause biochemical changes in the brain, which leads to behavioral changes in fish, as noted in the present study. The brains of fish directly exposed to nPS in the present study could also have been affected, causing changes in their activities and behaviors. These behavioral changes include reduced feeding activity or vitality of individuals^46,51^, which can inhibit growth and reproduction. Reduced ability to avoid other predators is also a serious problem that can affect the health of natural ecosystems, ecosystem services, and productivity of fisheries^51^.

In this study, we exposed algae (*C*. *reinhardtii*) and water fleas (*D*. *magna*) to higher nPS concentrations than those encountered in a natural environment, to assess the effects of nPS on freshwater organisms. High concentration levels were also used in the trophic transfer experiment, for indisputable visual confirmation of nPS transfer to organisms. The extremely high concentrations used in the dose-response and trophic transfer experiments rarely exist in real environmental conditions^57^. However, to evaluate and assess the potential impact of nanoplastics and the reaction of organisms to them, these types of experiments are needed, although the results should be considered with caution. Nevertheless, in the natural environment, organisms might be exposed to low concentrations of nanoplastics for a long time; thus, there is the potential for cumulative build-up in their bodies. Future studies should also consider, and attempt to simulate real environmental conditions to elucidate the present-day impact of nanoplastics in the environment^58^.

Additionally, because these small plastics reached a high level in the food chain, it is possible that nPS can be transferred to consumers at an even higher trophic level, like humans, through their diet. Additional studies on acute and chronic toxicity of micro- and nanoplastics are underway to investigate various effects and mechanisms. The acute and chronic toxicity of micro- and nanoplastics also require further study.

## Methods

### nPS

Fluorescent nPS (10 mg/mL) was purchased from Bangs Laboratories, Inc. (Fishers, IN, USA). According to the manufacturer, these fluorescent microspheres have a 480-nm excitation and 520-nm emission wavelength and a mean diameter of 51 nm. The stock solution (% in product) contained water (≥89.81%), polystyrene (≤10%), surfactant (≤0.1%), and sodium azide (≤0.09%). ζ-potentials and hydrodynamic diameter of nPS were measured via the dynamic light-scattering technique (Zetasizer Nano ZS, Malvern Instruments, UK) in distilled water (DW), moderately hard water (MHW; combinations of magnesium sulfate, sodium bicarbonate, potassium chloride, and hydrated calcium sulfate)^59^, and tris-acetate-phosphate medium (TAP; combinations of TAP salts, phosphate solution, and Hunter’s trace elements: zinc, boron, manganese, cobalt, copper, molybdenum, and iron)^60^. We identified the surface charges, conditions, and status of nPS in each medium and confirmed which nPS properties remained the same in different media for storage, cultivation, and testing. In addition, nPS particles were observed using a high resolution scanning electron microscope (HR-SEM; SU 70, Horiba, Kyoto, Japan).

### Test species

Trophic transfer was explored in a four-species freshwater ecosystem food chain consisting of *Chlamydomonas reinhardtii*, *Daphnia magna*, *Oryzias sinensis*, and *Zacco temminckii*. *C*. *reinhardtii* (UTEX, USA), a freshwater green alga with two flagella, was the primary producer in this experimental system. The cell size of *C*. *reinhardtii* was 2–20 μm. *D*. *magna*, a water flea that is the typical test species in ecotoxicity tests, was the primary consumer in this food chain. The body size of the neonate was 1.2–1.6 mm. *O*. *sinensis*, the Chinese rice fish, belongs to the same genus as the Japanese rice fish *O*. *latipes*, and has a body size of 25–30 mm. It was the secondary consumer in this food chain. *Z*. *temminckii* (dark chub), a large fish with a body size of 130–150 mm, was used as the end consumer. *C*. *reinhardtii* and *D*. *magna* were incubated in TAP (pH 7.1 ± 0.0) in a shaking incubator (24 ± 1 °C) and in MHW (pH 7.6 ± 0.3) in an incubator (21 ± 1 °C), respectively. Both incubators used a photoperiod of 16:8 h (light:dark). *O*. *sinensis* and *Z*. *temminckii* were purchased from an aquarium and fishery, respectively, and were acclimated in dechlorinated tap water for 2–4 weeks in the laboratory before the test. All experiments were performed in accordance with the regulations for ethical incubation, experimental use, and euthanasia of test animals, and all experimental protocols were approved by the Institutional Animal Care and Use Committee at Konkuk University, under authorization code number KU16096.

### Trophic transfer of nPS from *C. reinhardtii* to *Z. temminckii*

A total of three trophic transfer assays (*C*. *reinhardtii* → *D*. *magna*, 300 control-group and 300 exposed *D*. *magna*; *C*. *reinhardtii* → *D*. *magna* → *O*. *sinensis*, 30 control-group and 30 exposed *O*. *sinensis*; and *C*. *reinhardtii* → *D*. *magna* → *O*. *sinensis* → *Z*. *temminckii*, three control-group and three exposed *Z*. *temminckii*) were conducted, spanning a week, and the experiment was carried out three times (triplicate). Every fish in the test was visually observed and 10 *D*. *magna* in each treatment were observed. First, 1 mL of 1 × 10^7^ cells/mL *C*. *reinhardtii* was exposed to 50 mg/L nPS in a 1.75-mL test tube in an end-over-end rotator (LNS-MP8; Daihan Science, Korea) at 24 °C, with a 16:8 h (light:dark) photoperiod for 72 h. After this direct exposure, 1 mL of *C*. *reinhardtii* was washed with DW at 5,000 rpm for 1 min and then added to 50 mL of MHW in an 85-mL flat-bottomed glass vial (internal diameter, 40 mm; length, 75 mm) containing 100 neonates (<24 h) of *D*. *magna* for 5 h. Next, all alga-fed *D*. *magna* were collected and washed with MHW once, to remove adhered nPS. The *D*. *magna* were fed to 10 *O*. *sinensis* in 8 L of dechlorinated tap water in a glass tank (width, 0.2 m; length, 0.2 m; height, 0.25 m). These feedings occurred twice during 48 h. Finally, 10 *O*. *sinensis* that had been fed contaminated *D*. *magna* were washed with dechlorinated tap water and fed to a single *Z*. *temminckii* in 30 L of dechlorinated tap water in a plastic tank (width, 0.6 m; length, 0.4 m; height, 0.21 m). This feeding occurred once in a 24-h period, with four replicates.

Following trophic transfer tests, *C*. *reinhardtii* and *D*. *magna* were observed using an optical microscope (Olympus BX 51; Olympus, Japan) equipped with a fluorescent filter (excitation wavelength, 460–495 nm; emission wavelength, 510 nm; U-MWIB3; Olympus Cooperation, Tokyo, Japan). The green emission of nPS was observed, separated from the red emission of algal auto-fluorescence. Next, CLSM was used to observe control and exposed *C*. *reinhardtii* (LSM710; Carl Zeiss, Germany) and *D*. *magna* (FV-1000 spectral; Olympus, Japan). Confocal images of nPS attached to the surface of *C*. *reinhardtii* and within the guts of *D*. *magna* and Z-stack images (stacks of multiple images taken at different focal lengths) were analyzed. Z-stack images of *C*. *reinhardtii* were taken under specific conditions: entire depth 49 μm; 49 total images; each sheet 1 μm thick. Images of *D*. *magna* were taken under specific conditions: entire depth 236 μm; 236 total images; each sheet 1 μm thick. In addition, the gastrointestinal tract and microvilli of controlled and exposed *D*. *magna* were observed using a Bio-TEM (Tecnai G2SpiritTwin; FEI, USA) to confirm the effect of nPS on the organs.

*O*. *sinensis* and *Z*. *temminckii* exposed through the diet to nPS were also analyzed via an optical microscope with a fluorescent filter. *O*. *sinensis* and *Z*. *temminckii* were anesthetized and the intestines were removed. Because of the physical size and thickness of the fish, the digestive organs were extracted and the organs and contents were observed under the microscope. The intestines of *O*. *sinensis* were observed via CLSM (LSM710; Carl Zeiss, Germany) to determine whether nPS was present in the inner part of the organ. The Z-stack images of *O*. *sinensis* were taken under specific conditions: entire depth 219 μm; 219 total images; each sheet 1 μm thick. The excitation wavelengths of the CLSM laser were 488 and 543 nm for nPS fluorescence and for auto-fluorescence of algae, respectively. Unfortunately, the digestive organs of *Z*. *temminckii* could not be observed directly under the microscope because of their thickness and opacity. Instead, we dissected the organs, collected the inner contents of the intestine and stomach, and observed them via microscope to determine the amount of nPS transferred via the diet.

### Direct exposure of test species to nPS

We carried out individual toxicity tests of nPS on *C*. *reinhardtii* and *D*. *magna* using modified Test Guidelines No. 201^61^ and No. 202^62^, respectively, of the Organisation for Economic Co-operation and Development. The detailed protocol was adapted from Chae and An^63^. *C*. *reinhardtii* was exposed to a maximum 100 mg/L nPS (0, 20, 40, 60, 80, and 100 mg/L), and *D*. *magna* was exposed to a maximum 10 mg/L nPS (0, 2, 4, 6, 8, and 10 mg/L). After exposure, chlorophyll fluorescence of *C*. *reinhardtii* was measured via a fluorescence microplate reader (GeminiEM; Molecular Devices, USA). The mortality and immobilization of *D*. *magna* were quantified at 24 and 48 h.

The direct exposure experiments on *O*. *sinensis* and *Z*. *temminckii* were carried out once. A total of eight *O*. *sinensis* (including embryos that hatched during the experiment) and three *Z*. *temminckii* were exposed to nPS at 5 mg/L in aerated glass tanks individually (one *Z*. *temminckii* in the exposed group escaped from the glass tank and died). Liver histopathology, the amount of cholesterol in blood serum, embryonic uptake, and locomotive activity were analyzed. After 7 d of exposure, the livers of *Z*. *temminckii* were extracted and liver histopathology was analyzed after dehydrating, sectioning, and hematoxylin and eosin (H&E) staining the nuclei of cells (acidic, negatively charged) and other structures (positively charged amino acid side chains). The cholesterol contents in the blood serum of *Z*. *temminckii* were also investigated using an HDL and LDL/VLDL cholesterol assay kit (Catalog # ab65390, Abcam, Cambridge, MA).

*O*. *sinensis* spawned during the exposure and the fertilized eggs were exposed to nPS for 24 h. The eggs were then transferred into an embryo-rearing solution and incubated at room temperature for microscopic analysis. Embryonic uptake in *O*. *sinensis* was observed, using an optical microscope equipped with a fluorescent filter, 5 d after transfer.

The locomotive activities of both *O*. *sinensis* and *Z*. *temminckii* were observed in a tank (30 cm wide × 20 cm long × 20 cm tall) containing 10 L dechlorinated water. Non-exposed and exposed fish were placed in tanks containing clean tap water but no food (four control *O*. *sinensis*, four exposed *O*. *sinensis*, three control *Z*. *temminckii*, and two exposed *Z*. *temminckii* in each tank). After allowing 5 min for acclimation, their activities were recorded using a digital camera. The videos were recorded for 30 s with images captured at 0.3-s intervals. These 100 images were merged and analyzed by placing dots on the captured images via Adobe Photoshop, and the area and distances traveled were investigated using Image J (free software). Because of the small number of individuals, these recordings were considered pseudoreplication.

### Statistical analysis

We statistically analyzed the results of individual toxicity tests of nPS on growth in *C*. *reinhardtii*, survival of *D*. *magna*, and behavior of *O*. *sinensis* (analysis on *Z*. *temminckii* was excluded because of the lack of replicates). To confirm the reliability of the data, we first checked its normal distribution and the homoscedasticity of its variance in Origin pro 8.0 (OriginLab Corp., MA, USA). One-way analysis of variance (ANOVA) followed by the Dunnett^64^ t-test (two-tailed post-hoc) for multiple comparisons was performed using EMSL Cincinnati Dunnett 1.5, to identify significant differences between the control and nPS-exposed groups. A 95% significance level (*p* < 0.05) was used for all comparisons.

## Electronic supplementary material

Supplimentary Information
