# Supplementary material for: Trophic transfer and individual impact of nano-sized polystyrene in a four-species freshwater food chain
Source: Sci Rep. 2018 Jan 10;8:284. doi: 10.1038/s41598-017-18849-y (PMC5762726; doi:10.1038/s41598-017-18849-y)
Supplement: Supplementary file 1 — Supplimentary Information [file 41598_2017_18849_MOESM1_ESM.pdf]

Supplementary Information

Trophic transfer and individual impact of nano-sized polystyrene in a four-species freshwater food chain

Yooeun Chae, Dokyung Kim, Shin Woong Kim & Youn-Joo An*

Department of Environmental Health Science, Konkuk University, 120 Neungdong-ro, Gwangjin-gu, Seoul 05029, Korea

*** Corresponding author**
Youn-Joo An
Telephone: +82-2-2049-6090 | Fax: +82-2-2201-6295 | E-mail: anyjoo@konkuk.ac.kr

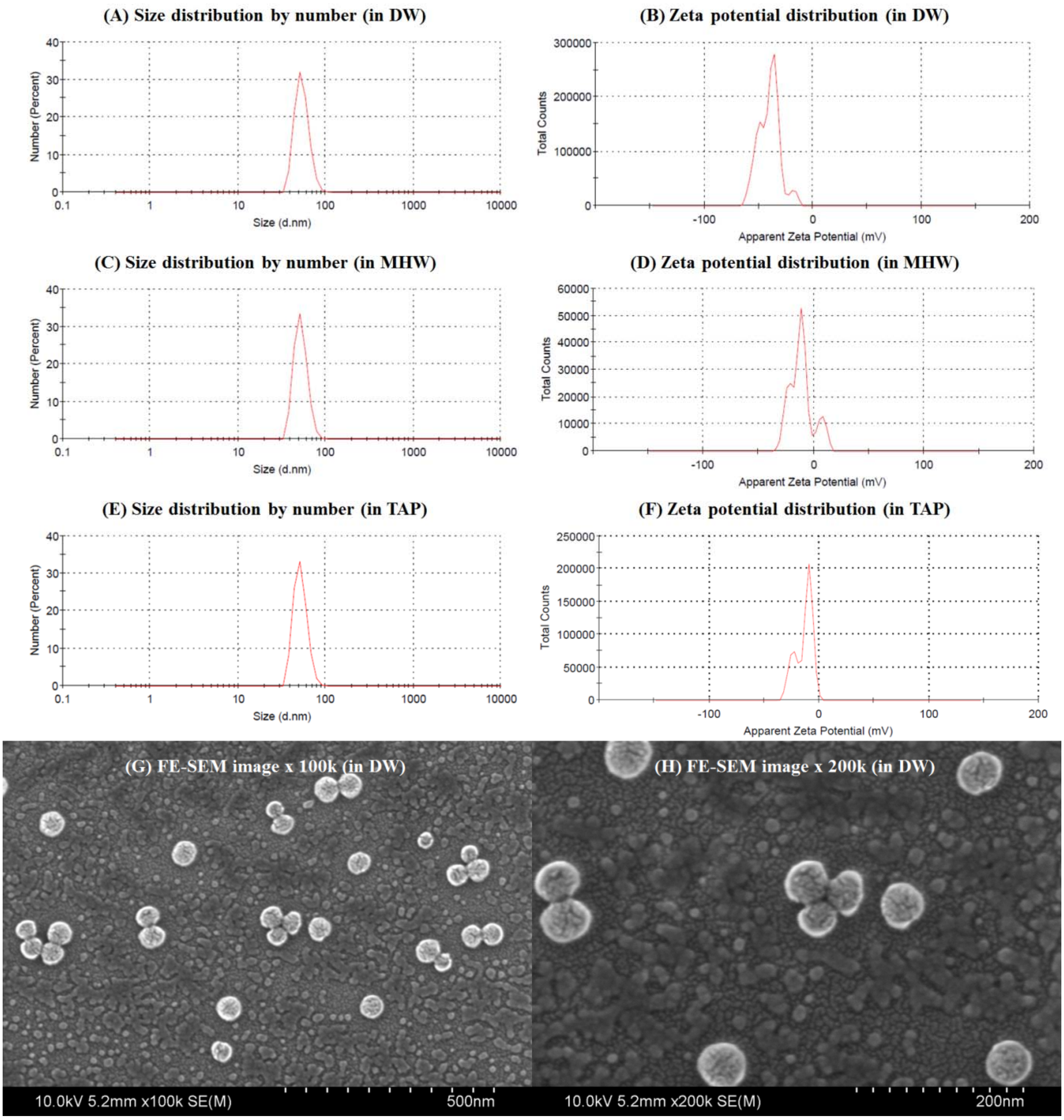

Supplementary Figure 1. Size distribution and ζ-potential of nano-sized polystyrene (nPS) in distilled water (DW) (A, B), moderately hard water (MHW) (C, D) and tris-acetate-phosphate medium (TAP) (E, F). Images of fluorescent nPS were taken using a field-emission scanning electron microscope (FE-SEM) (G, H).

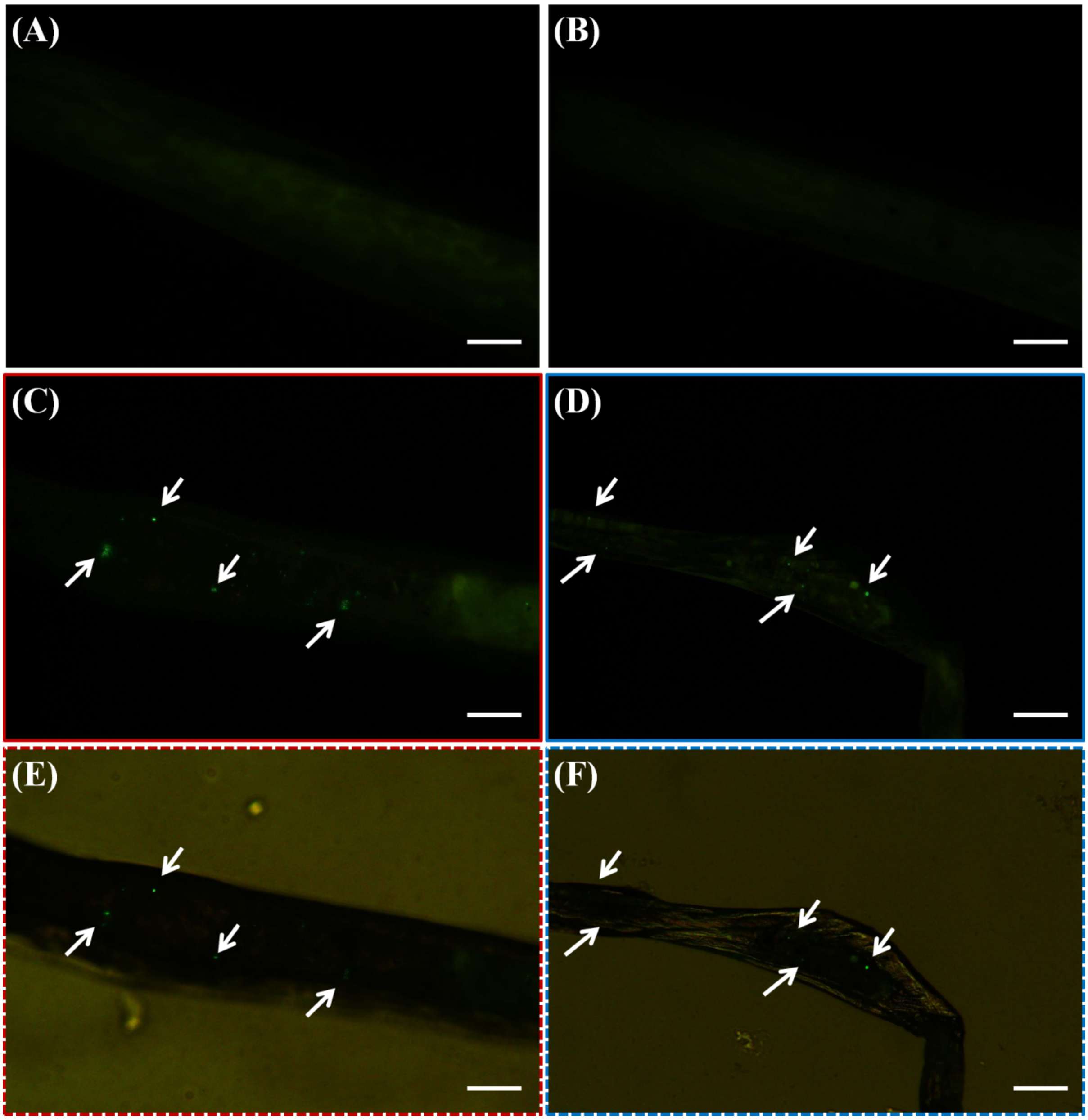

Supplementary Figure 2. Fluorescent images of *Oryzias sinensis* intestine. Control groups (A, B) were not exposed to nano-sized polystyrene (nPS), while exposed groups (C, D) were fed contaminated *Daphnia magna*. E and F are merged (bright field and fluorescent) images of C and D, respectively. White arrows indicate fluorescent nPS. Scale bar = 200 μm.

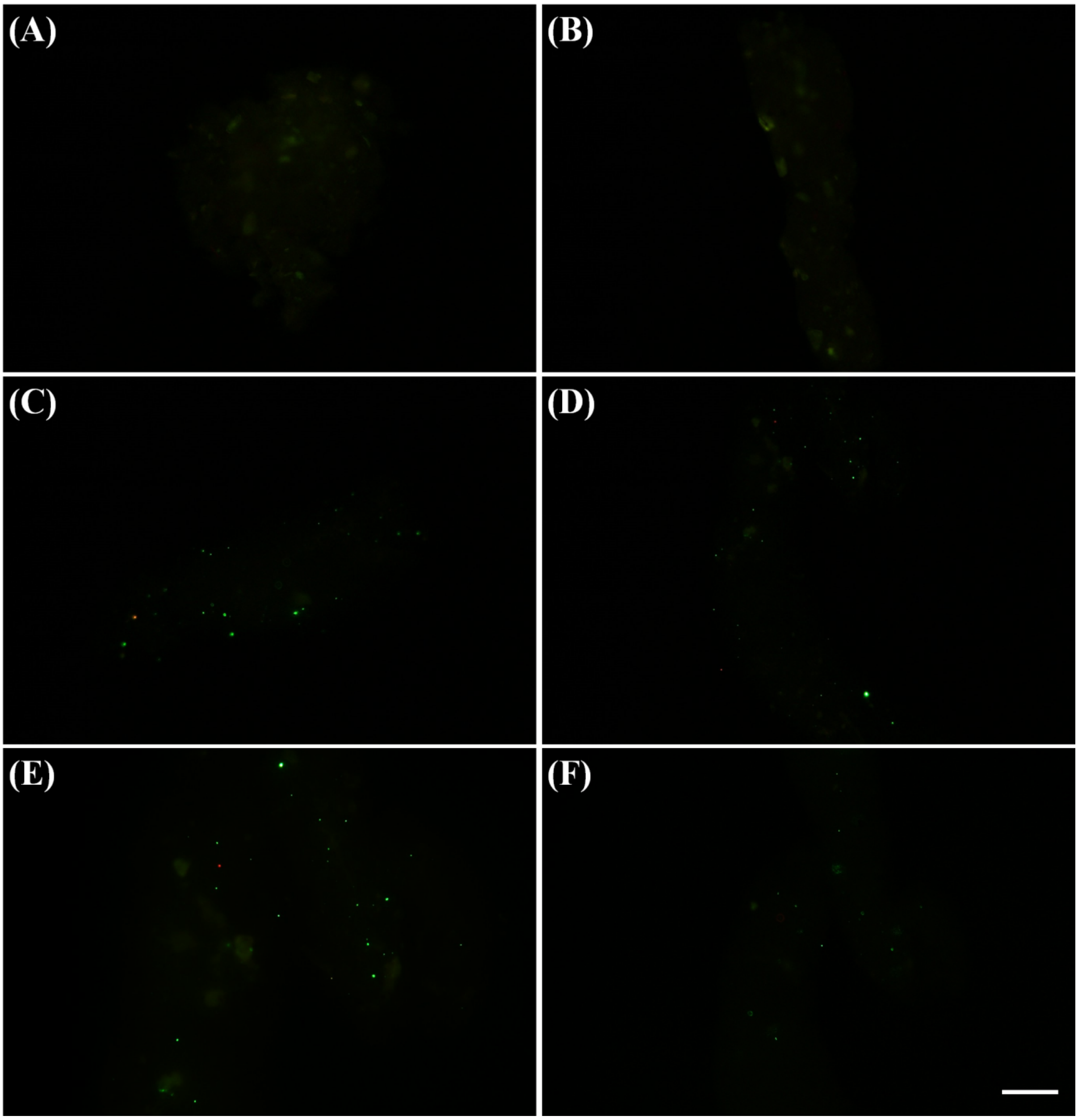

Supplementary Figure 3. Feces of *Oryzias sinensis*. Control groups (A, B) were not exposed to nano-sized polystyrene (nPS), while exposed groups (C–F) were fed contaminated *Daphnia magna* for 48 h. Scale bar = 200 μm.

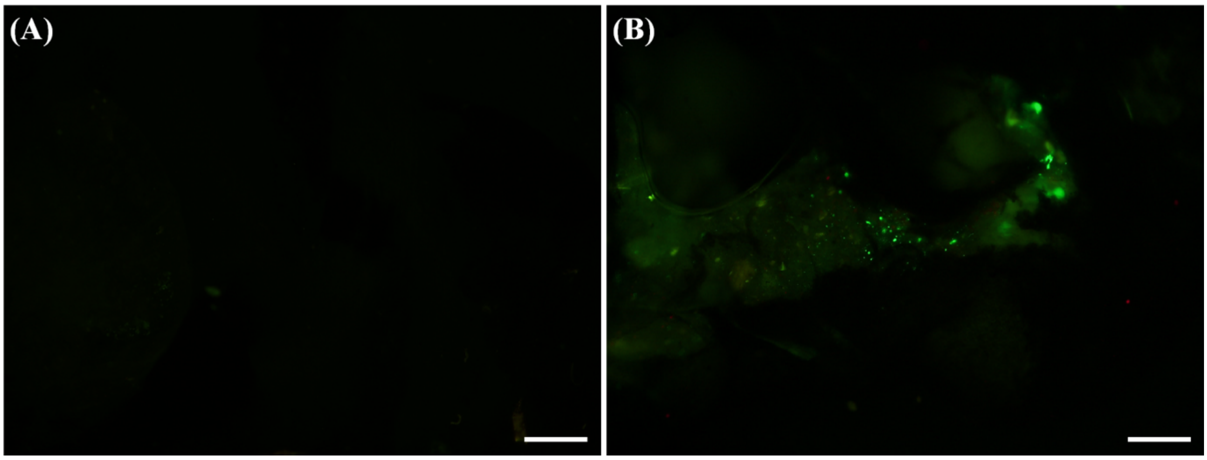

Supplementary Figure 4. The feces of *Zacco temminckii*. Green fluorescence indicating nano-sized polystyrene (nPS) was not observed in the control group (A) but was apparent in the exposed group (B), exposed through the diet to nPS for 24 h. Scale bar = 200 μm.

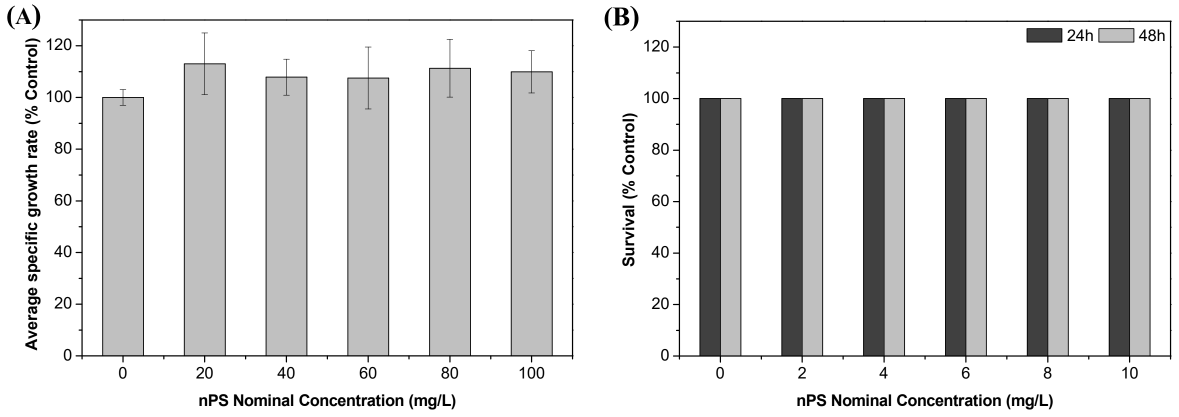

Supplementary Figure 5. The individual toxicity of nano-sized polystyrene (nPS) (51-nm diameter) on (A) *Chlamydomonas reinhardtii* ($p > 0.05$) and (B) *Daphnia magna* ($p > 0.05$).

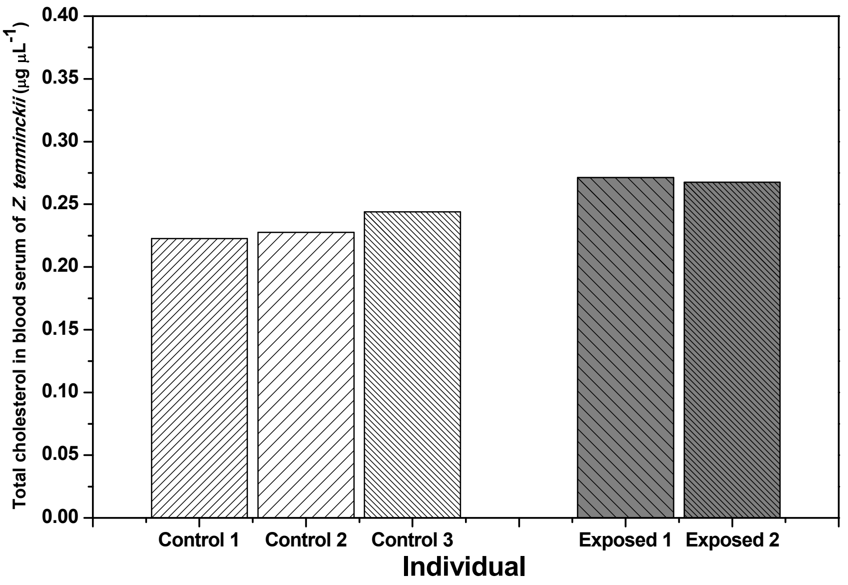

Supplementary Figure 6. Total amount of cholesterol in blood serum of *Zacco temminckii* from control group (C1–C3) and exposed group (E1–E2). Each column represents the amount of total cholesterol in each individual.
